# Color Discrimination in the Tufted Capuchin Monkey, *Sapajus* spp

**DOI:** 10.1371/journal.pone.0062255

**Published:** 2013-04-19

**Authors:** Paulo Roney Kilpp Goulart, Daniela Maria Oliveira Bonci, Olavo de Faria Galvão, Luiz Carlos de Lima Silveira, Dora Fix Ventura

**Affiliations:** 1 Núcleo de Teoria de Pesquisa do Comportamento, Universidade Federal do Pará, Belém, Pará, Brazil; 2 Departamento de Psicologia Experimental, Instituto de Psicologia, Universidade de São Paulo, São Paulo, Brazil; 3 Núcleo de Neurociências e Comportamento, Universidade de São Paulo, São Paulo, São Paulo, Brazil; 4 Núcleo de Medicina Tropical, Universidade Federal do Pará, Belém, Pará, Brazil; 5 Instituto de Ciências Biológicas, Universidade Federal do Pará, Belém, Pará, Brazil; Monash University, Australia

## Abstract

The present study evaluated the efficacy of an adapted version of the Mollon-Reffin test for the behavioral investigation of color vision in capuchin monkeys. Ten tufted capuchin monkeys (*Sapajus* spp., formerly referred to as *Cebus apella*) had their DNA analyzed and were characterized as the following: one trichromat female, seven deuteranope dichromats (six males and one female), and two protanope males, one of which was identified as an “ML protanope.” For their behavioral characterization, all of the subjects were tested at three regions of the Commission International de l'Eclairage (CIE) 1976 u′v′ diagram, with each test consisting of 20 chromatic variation vectors that were radially distributed around the chromaticity point set as the test background. The phenotypes inferred from the behavioral data were in complete agreement with those predicted from the genetic analysis, with the threshold distribution clearly differentiating between trichromats and dichromats and the estimated confusion lines characteristically converging for deuteranopes and the “classic” protanope. The discrimination pattern of the ML protanope was intermediate between protan and deutan, with confusion lines horizontally oriented and parallel to each other. The observed phenotypic differentiation confirmed the efficacy of the Mollon-Reffin test paradigm as a useful tool for evaluating color discrimination in nonhuman primates. Especially noteworthy was the demonstration of behavioral segregation between the “classic” and “ML” protanopes, suggesting identifiable behavioral consequences of even slight variations in the spectral sensitivity of M/L photopigments in dichromats.

## Introduction

The first behavioral studies on color vision in New World monkeys (platyrrhine primates) were performed with capuchin monkeys at the end of the 1930s [1], [2]. The three male capuchins in these studies that were subjected to experiments on wavelength discrimination, color matching, and neutral points of spectral sensitivity showed reduced sensitivity at the long-wavelength end of the spectrum compared with humans and other catarrhine primates (i.e., Old World monkeys) evaluated with similar tests. This finding seemed to indicate that capuchin monkeys had color vision phenotypes that were similar to those of protanopic humans and that the New World monkeys could be used to study intermediate evolutionary stages of trichromatic color vision.

Additional behavioral studies and biological investigations with other New World monkeys, especially squirrel monkeys (*Saimiri* sp.), demonstrated the occurrence of intraspecies phenotypic variations [3]–[5]. Electrophysiological [4]–[8] and genetic [9], [10] investigations documented the occurrence of multiple M/L photopigments in platyrrhine monkeys related to the presence of a single polymorphic gene on the X chromosome that encodes M/L photopigments. According to the resulting model, New World monkeys have a sex-linked polymorphic gene for color vision, with some females being trichromats and all of the males and the remainder of females being dichromats. In these polymorphic species, the X chromosome possesses a single gene that is responsible for the expression of cone photopigments, with multiple allelic versions that occur in the same species [11]–[14]. Each allele is responsible for minute variations in the amino acid sequence that composes the protein portion (opsin) of the photopigment. These variations, in turn, translate to differences in photopigment sensitivity along the medium- to long-wavelength region of the light spectrum. Similar genotype/phenotype arrangements have been observed in the majority of the New World monkeys studied to date, including capuchin monkeys [10], [15], [16].

Three variants of medium/long-wavelength-sensitive photopigments have been consistently described for capuchin monkeys, with spectral peaks near 530, 550, and 560 nm [14], herein referred to as M, ML, and L photopigments, respectively. The different combinations of short-wavelength (S)-sensitive photopigments and medium/long-wavelength-sensitive photopigments produce six different color vision phenotypes within the same population. Heterozygous females (i.e. those that have a different allele in each of their X chromosomes) express two different medium/long wavelength-sensitive photoreceptors. Depending on the specific alleles present, each individual shows one of three trichromatic phenotypes (S-M-L, S-M-ML, or S-ML-L). In contrast, homozygous females and males express only one of the three possible medium/long-wavelength-sensitive cone photoreceptors. Each individual then shows one of three dichromatic phenotypes (S-M, S-ML, S-L).

Decades of accumulated knowledge on the interactions among opsin genes, photopigments, and the behavioral expression of color vision in New World monkeys has made the prediction of color discrimination capabilities of these animals based on their anatomical and physiological attributes a common practice. Monkeys that possess two cone opsins (all males and homozygous females) are expected to show impaired color discrimination, characterized by deutan or protan loss, depending on the relative sensitivity to medium/long wavelength components. Additionally, only heterozygous females that possess M/L wavelength-sensitive cones of two types, with sufficiently spaced peak sensitivities, are predicted to show color discrimination performance that is similar to normal trichromatic humans [17]. However, for the majority of species, little or no direct behavioral evidence of the potential or limitations of color discrimination has been provided.

Concerning specifically capuchin monkeys, the occurrence of trichromatic color vision in some females and dichromatic color vision in males and the remainder of females is supported by electrophysiological studies of photopigments, in which only one class of M/L cones was found in the retina in males [8], [15], and DNA analyses that confirmed the presence of a single opsin gene on the X chromosome [8], [16].

Over many years, a comprehensive description of the retina and visual system of capuchin monkeys has been derived from electrophysiological studies [8], [18]–[24]. To date, the morphology and distribution of ganglion cells [21], [25]–[30], bipolar cells [31], [32], horizontal cells [33], rods, and cones [34]–[37] have been extensively characterized.

At this point, an observation is in order regarding the omission of scientific names when capuchin monkeys were mentioned above. Until recently, tufted and untufted (also known as robust and gracile) capuchin monkeys were both placed in the genus *Cebus.* Tufted capuchins were broadly referred to as *Cebus apella*, despite the existence of competing taxonomic hypotheses recognizing the diversity of the “apella” group. An examination of morphological, genetic, behavioral, ecological, and biogeographic evidence [38] indicated that the tufted and untufted lineages differentiated from a common ancestor approximately 6.2 million years ago. Based on this finding, tufted capuchins are now grouped under a separate genus, *Sapajus*, and are thought to comprise at least seven species. Since most of the previous research on color vision in “*Cebus* monkeys” was performed with tufted capuchins, the scientific names were deliberately omitted in order to avoid confusion. It remains to be established whether the findings from those studies can indeed be extrapolated to actual *Cebus* monkeys (i.e., untufted capuchins).

The objective of the present study was to evaluate the efficacy of an adapted version of the Mollon-Reffin test developed by Goulart et al. [39] for the detailed characterization of color discrimination phenotypes of tufted capuchin monkeys (*Sapajus* spp.). Similar to the adaptation developed by Mancuso et al. [40] for squirrel monkeys (*Saimiri* sp.), a critical modification was the change of the shape of the target stimulus to a square that could appear at multiple locations on the screen and should be touched by the subjects. Although the Mollon-Reffin test is based on the Commission International de l'Eclairage (CIE) chromaticity diagram, derived from and for human observers, Mancuso et al. argued that it could be used with squirrel monkeys because the spectral sensitivity of their M/L wavelength-sensitive photopigments are similar to equivalent photopigments in humans. The same argument supports its use with tufted capuchin monkeys because the spectral sensitivity of their M/L photopigment variants is similar to squirrel monkeys [14].

The phenotypes inferred from the behavioral data should match those predicted from the genetic analysis of the opsin genes possessed by each individual. Similar to human subjects, dichromatic animals were expected to show poor discrimination in at least two complementary test vectors, corresponding to dominant wavelengths that are indistinguishable from the background [40]. When plotted on the CIE chromaticity diagram color discrimination thresholds in dichromatic animals were expected to have an ellipsoid distribution, with one axis considerably longer than the other, whereas color discrimination thresholds in trichromatic animals could be expected to show a smaller area and an approximately circular distribution.

## Materials and Methods

### Ethics statement

Protocols for the general care of the animals were approved by the Brazilian Institute for the Environment and Renewable Natural Resources (IBAMA, Instituto Brasileiro do Meio Ambiente e dos Recursos Naturais Renováveis) according to local and international ethical guidelines. All of the experiments were conducted in accordance with National Institutes of Health guidelines regarding the care and use of animals for experimental procedures. The housing, handling, feeding and veterinary care, as well as the specific experimental procedures adopted, were approved by the Animal Research Ethics Committee (CEPAE) of the Federal University of Pará (document code CEPAE-UFPA PS001/2005). No monkeys were sacrificed during the study.

### Subjects

Ten tufted capuchin monkeys (*Sapajus* spp.), eight males and two females, with a previous history of behavioral discrimination procedures, were used. The subjects are housed in groups of 3–4 animals in cages that measure 2.50 m×2.50 m×2.50 m, located in the external courtyard of the Experimental School for Primates, in the Behavior Theory and Research Center of the Federal University of Pará. The cages are exposed to environmental lighting variations that are characteristic of the region, with a cycle of approximately 12 h daylight and 12 h dark, disposed in the East-West axis. Each cage is 1.50 m apart from each other, placed on a cement floor and under a continuous roof that covers the Northern half of each cage so that it blocks direct sun light in part of its interior. The interior of the nursery cages is rich in walkways, shelters, several objects for manipulation, and some apparatuses developed to simulate tool use and foraging. Each nursery cage has four lateral compartments used for isolation and/or feeding, that have sliding doors with locks and a transfer cage, which allows withdrawal of the animals using a transportation cage. The animals are maintained in health and feeding conditions approved by IBAMA, under supervision of a medical veterinary. The animals have free access to water and receive a balanced daily diet based on fruits, vegetables and protein. Feeding occurs once a day (between 15:00 and 16:00) and cage cleaning is conducted during the morning, approximately and 7:00. No additional deprivation is used.

### Collection of blood samples

For the collection of blood samples, the animals were sedated with anesthetic association of tiletamine chloride and zolazepam chloride (10 mg/kg). The blood was collected from the femoral vein with a 22G hypodermic needle adapted to a vacutainer tube containing sodium citrate. After the blood collection, for security reasons, the animals were placed in enclosure cages into the housing cages until they returned to normal activities.

### Genetic analysis

Genetic analysis was performed at the Laboratory of Sensory Psychophysiology, in the Institute of Psychology of the University of São Paulo. DNA was extracted from blood samples using a purification kit (Puregene DNA, Gentra System). Polymerase chain reaction (PCR) was performed as described by Mancuso et al. [40] to amplify exons 3 and 5 of the X-linked opsin genes. The sequences of the forward and reverse primers for exon 3 were 5′-GGATCACGGGTCTCTGGTC and 5′-CTGCTCCAACCAAAGATGG, respectively. For exon 5, the forward primer was 5′-GTGGCAAAGCAGCAGAAAG, and the reverse primer was 5′-CTGCCGGTTCATAAAGACATAG. The PCR products were directly sequenced using the DYEnamic ET Dye Terminator kit on a MegaBACE 1000 sequencer (GE Healthcare). The amino acid sequences from exons 3 and 5 were used to estimate the maximum absorption peak of the pigment within a range of values identified by Asenjo et al. [41] for variant opsins containing the same amino acids at those positions. Phenotypic classification followed Soares et al. [42].

### Behavioral tests

#### Apparatus

The training and testing parameters were controlled by an adapted version of the Mollon-Reffin test paradigm (developed in Object Pascal by Marcio L. Bandeira, Paulo R.K. Goulart, Nestor N. Oiwa, Marcelo F. Costa, and Dora F. Ventura). Stimuli were generated by the ViSaGe system (Cambridge Research Systems, Rochester, UK) and displayed on a high-performance cathode-ray tube (CRT) monitor (Diamond Pro 2070SB, Mitsubishi, Cypress, CA, USA). The monitor was calibrated on a quarterly basis using an OptiCAL 200-E photometer (Cambridge Research Systems, Rochester, UK) and the standard calibration routine of the VSG Desktop library (version 8.0). The monitor was kept 20 cm from the subject using a wooden structure attached to the opening of the experimental chamber. The part of this structure to which the monitor was juxtaposed was equipped with a CarrolTouch Infrared (Elo TouchSystems, Menlo Park, CA, USA) touch-sensitive interface. A 190 mg pellet dispenser (Med Associates, St. Albans, VT, USA) provided one pellet per correct response. The experimental room was kept at a temperature of 22°C.

#### Stimulus

**Figure 1:**
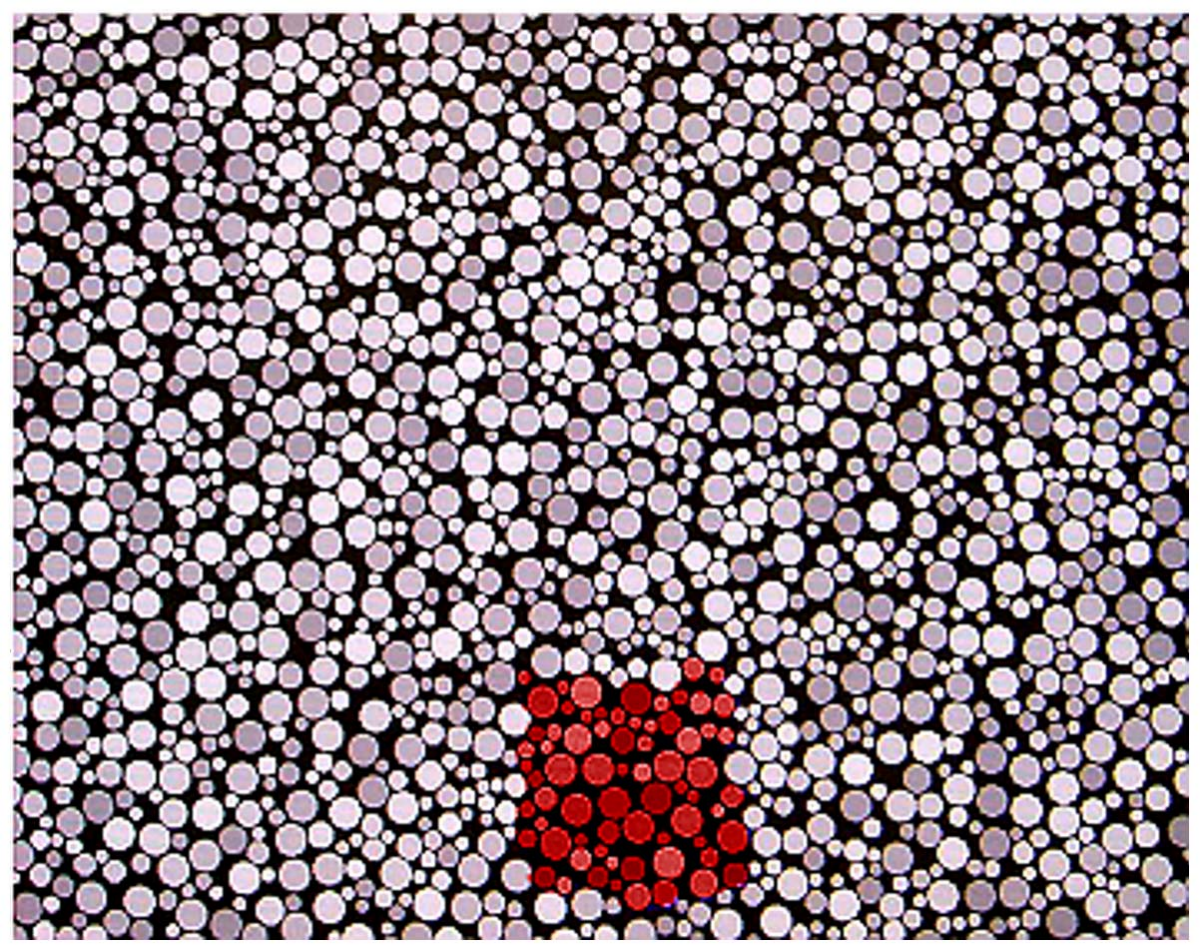
Example of the stimulus arrangement adopted in the present study. A pseudoisochromatic stimulus composed of circles of varying sizes and luminance is shown. The approximately square area with a different hue from background is the target.

The stimulus arrangement, occupying the entire screen, was composed of circles of different sizes at six levels of luminance that randomly varied between 7 and 15 cd/m^2^. The target was composed of a subset of circles that formed an approximately 5 cm^2^ square patch with a different hue from that of the remaining circles (Figure 1) and could appear at any of nine positions in a 3×3 matrix. Stimulus chromaticities were calculated in the CIE 1976 u′v′ diagram. The CRT phosphor limits were the following: red phosphor (R; u′ = 0.416, v′ = 0.522), green phosphor (G; u′ = 0.117, v′ = 0.559), and blue phosphor (B; u′ = 0.159, v′ = 0.177). The stimulus changed dynamically from trial to trial, with the target hue varying within and between chromatic variation vectors. The excursion along each vector could vary between 1100×10^-4^ and 20×10^-4^ u′v′ units.

#### General procedure

The duration of the experimental sessions was set to a maximum of 30 min, consisting of one or two blocks of trials. In each block, the trials continued to be generated according to the programmed parameters until the criterion was reached or until 100 trials or 10 min elapsed without a clear tendency of change in performance. Each trial began with the presentation of the stimulus and ended with its disappearance when a touch response was made at any point on the screen. The intertrial interval (ITI) was initially defined as 5 s and then adjusted according to the demands of each subject. Touch responses made on the target area were followed by the presentation of a 190-mg banana pellet and the ITI. Touch responses made on any other part of the screen were followed by the ITI alone.

#### Training phase

To bring the touch response under the control of the chromatic difference, the background chromaticity was defined at the achromatic point of the CIE 1976 diagram (u′ = 0.1977, v′ = 0.4689), and target chromaticities were defined at points outside any confusion lines predicted for the dichromats and presented in high saturation. Initially, the target-background distance was kept constant at 1100×10^−4^ u′v′ units. When the animals consistently touched the target, independent of its position, in three consecutive trials of each training vector in at least two consecutive blocks, the dynamic variation of the target-background distance was introduced, with the minimum distance of excursion along the vector defined as 500×10^−4^ u′v′ units. The criterion was then defined as three consecutive correct selections of the target at the minimum distance of each training vector in at least two consecutive blocks. When this criterion was reached, the minimum distance was redefined as 200×10^−4^ u′v′ units. When the criterion was again reached, the training was considered successful, and the animals were subjected to a pretest session that simulated the test conditions. In the test simulation, the animals were exposed to three novel vectors, and the minimum distance was set at 20×10^−4^ u′v′ units, a condition in which the animal would eventually fail to distinguish the target from the background, which is necessary for the calculation of discrimination thresholds. The animals were considered ready for the test phase if the target continued to control their behavior despite the unfamiliar hues, and they continued to respond under the control of the chromatic difference between the target and background even in low-contrast trials.

#### Testing phase

**Figure 2:**
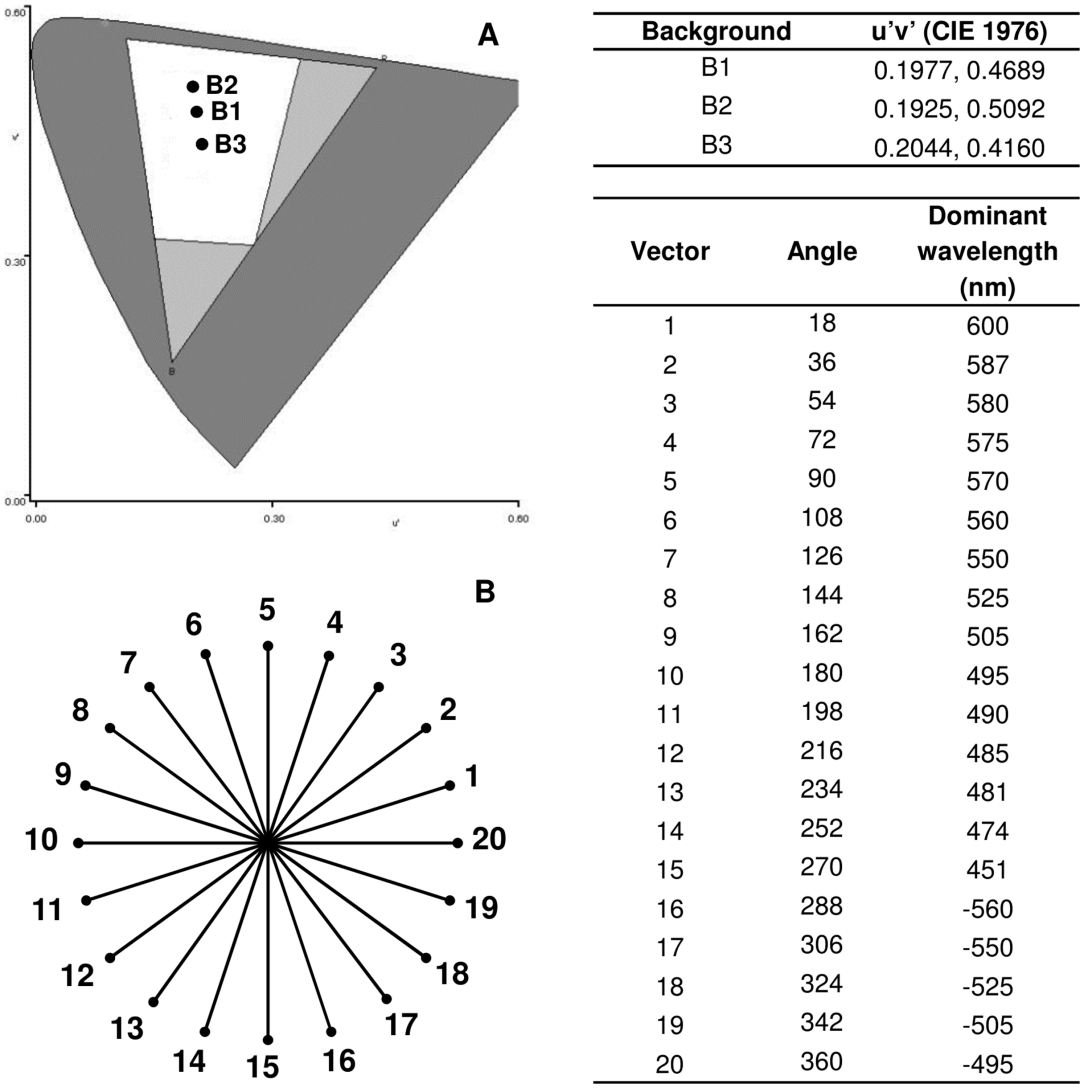
Testing phase parameters. (A) Chromaticity points used as background. (B) Distribution of the 20 test vectors. The CIE 1976 u′v′ coordinates for each background chromaticity are presented. Also shown are the angle and dominant wavelength for each vector tested from the achromatic point (Background 1) of the CIE 1976 u′v′ chromaticity diagram. Negative values refer to vectors that contain “purples and magentas,” which are nonspectral colors and have no dominant wavelength but are described by their complementary dominant wavelength, lying on the opposite side of the diagram.

Each subject was exposed to three tests composed of 20 test vectors for three different backgrounds, defined at the same background coordinates used by the Cambridge Colour Test (Cambridge Research Systems, Rochester, UK; Figure 2). Each test consisted of five sessions, each presenting four of the 20 vectors. The maximum distance of excursion along the vectors was 1100×10^−4^ u′v′ units, and the minimum distance was 20×10^−4^ u′v′ units. The pattern of correct and incorrect responses served as the basis for the determination of discrimination thresholds. When the subjects' performance reached the criterion of 11 reversal trials (correct followed by incorrect or incorrect followed by correct), a discrimination threshold was derived from the average distances between the background point and the target point at which the last seven reversals occurred. The distribution of the discrimination thresholds guided the behaviorally based phenotypic classification.

Data deposited in the Dryad repository: http://dx.doi.org/10.5061/dryad.k9f7t. Video recordings of the behavioral sessions will be made available upon request.

## Results and Discussion

### Genetic analysis

**Figure 3:**
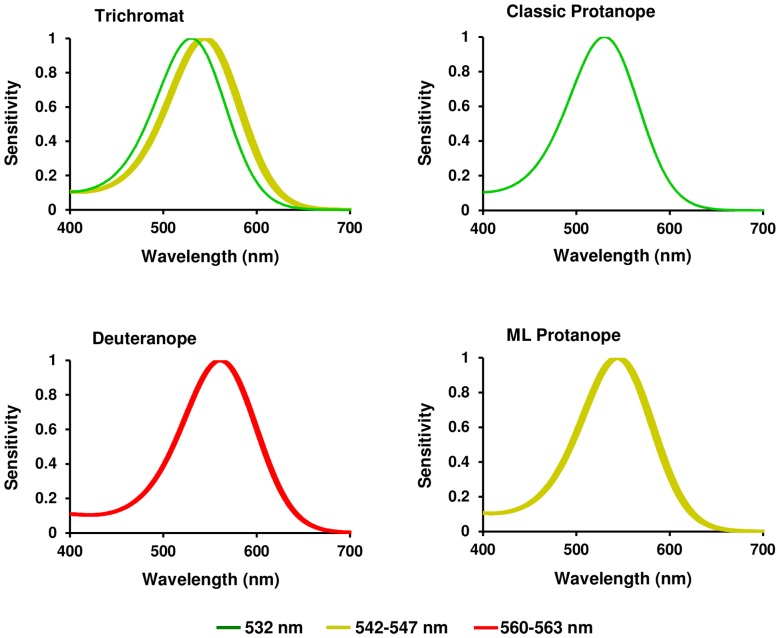
Spectral sensitivity curves of L and M cone pigments from capuchin monkeys . The maximum absorption peak of the visual pigments were predicted from the amino acid sequences from exons 3 and 5 of the X-linked opsin.

The genetic sequencing of exons 3 and 5 allowed the deduction of the amino acid combinations at positions 180, 277, and 285 of the resulting opsins, from which the range of potential absorption maxima of the corresponding photopigments was inferred (Figure 3). Female F1 was identified as a trichromat, presenting the amino acid combinations alanine-phenylalanine-alanine (AFA) and alanine-phenylalanine-threonine (AFT), corresponding to peaks at 532 nm and 542–547 nm, respectively. Six of the males and female F2 were identified as deuteranope dichromats, with an absorption peak at 560–563 nm, inferred from the amino acid combination serine-tyrosine-threonine (SYT). The other two males presented different amino acid combinations. M7 showed the combination AFA (absorption peak at 532 nm) and was characterized as a “classic protanope” dichromat, and M8 showed the combination AFT (absorption peak at 542–547 nm) and was characterized as an “ML protanope” dichromat according to Soares et al. (Table 1).

**Table 1:** Genotype, absorption peak, and phenotype for each monkey.

Subject	Sex	Genotype	λmax (nm)	Phenotype
F1	Female	AFA/AFT	532/542–547	Trichromat
F2	Female	SYT	560–563	Deuteranope dichromat
M1	Male	SYT	560–563	Deuteranope dichromat
M2	Male	SYT	560–563	Deuteranope dichromat
M3	Male	SYT	560–563	Deuteranope dichromat
M4	Male	SYT	560–563	Deuteranope dichromat
M5	Male	SYT	560–563	Deuteranope dichromat
M6	Male	SYT	560–563	Deuteranope dichromat
M7	Male	AFA	532	“Classic” protanope dichromat
M8	Male	AFT	542–547	“ML” protanope dichromat

Genotypes are identified by the amino acid combinations at positions 180, 277, and 285 of the opsin, AFA (alanine-phenylalanine-alanine), AFT (alanine-phenylalanine-threonine), and SYT (serine-tyrosine-threonine). The corresponding absorption peaks and phenotypic classification were determined based on Asenjo et al. (1994) and Soares et al. (2010), respectively.

### Behavioral tests

#### Training phase

All of the subjects successfully completed the training phase, consistently identifying the target in the trials that presented the minimum target-background contrast (minimum distance of 200×10^−4^ u′v′ units). At the end of training, the subjects were able to continue responding under the control of the chromatic difference, even when the contrast was very subtle, an essential precondition for exposure to the testing phase. When exposed to the test simulation, all of the subjects presented the necessary behavioral prerequisites for the calculation of thresholds during the test. Control by the target was maintained independently of the specific hue and persisted even in low-contrast trials. In fact, the animals responded consistently even in trials that were more difficult than those experienced during training (i.e., even when the distance was less than 200×10^−4^ u′v′ units).

#### Testing phase

**Figure 4:**
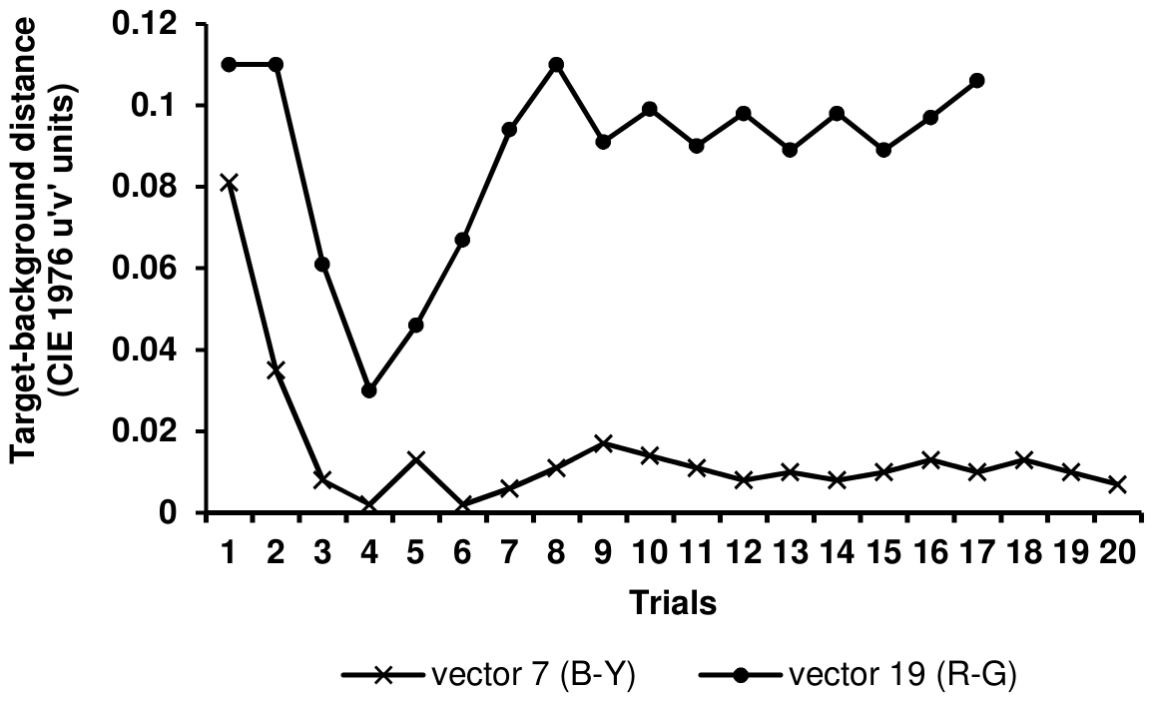
Example of staircases leading to threshold calculation. The figures shows the sequences produced by a male monkey in a blue-yellow oriented vector (7) and red-green oriented vector (19). Each point represents the distance, in u′v′ units, between the chromaticity points that define the target and background. The distance was decreased following correct responses and increased following incorrect responses.

Figure 4 presents two example sequences in a male monkey that led to thresholds in a blue-yellow-oriented vector (7) and red-green-oriented vector (19). Each point represents the distance, in u′v′ units, between the chromaticity points that defined the target and background. The distance was decreased following correct responses and increased following incorrect responses. Notably, because the animals were forced to touch the monitor in trials that presented indistinguishable target-hues, spurious correct responses eventually occurred, which could be observed in trials 3 and 4 of the sequence that corresponded to vector 19. If many such false positives occurred in the same vector, then the threshold could be underestimated. Similarly, some spurious mistakes could also occur (results not shown) because the monkeys touched near the periphery of the target. Such false negatives would overestimate the thresholds in vectors for which good discriminability was expected.

**Figure 5:**
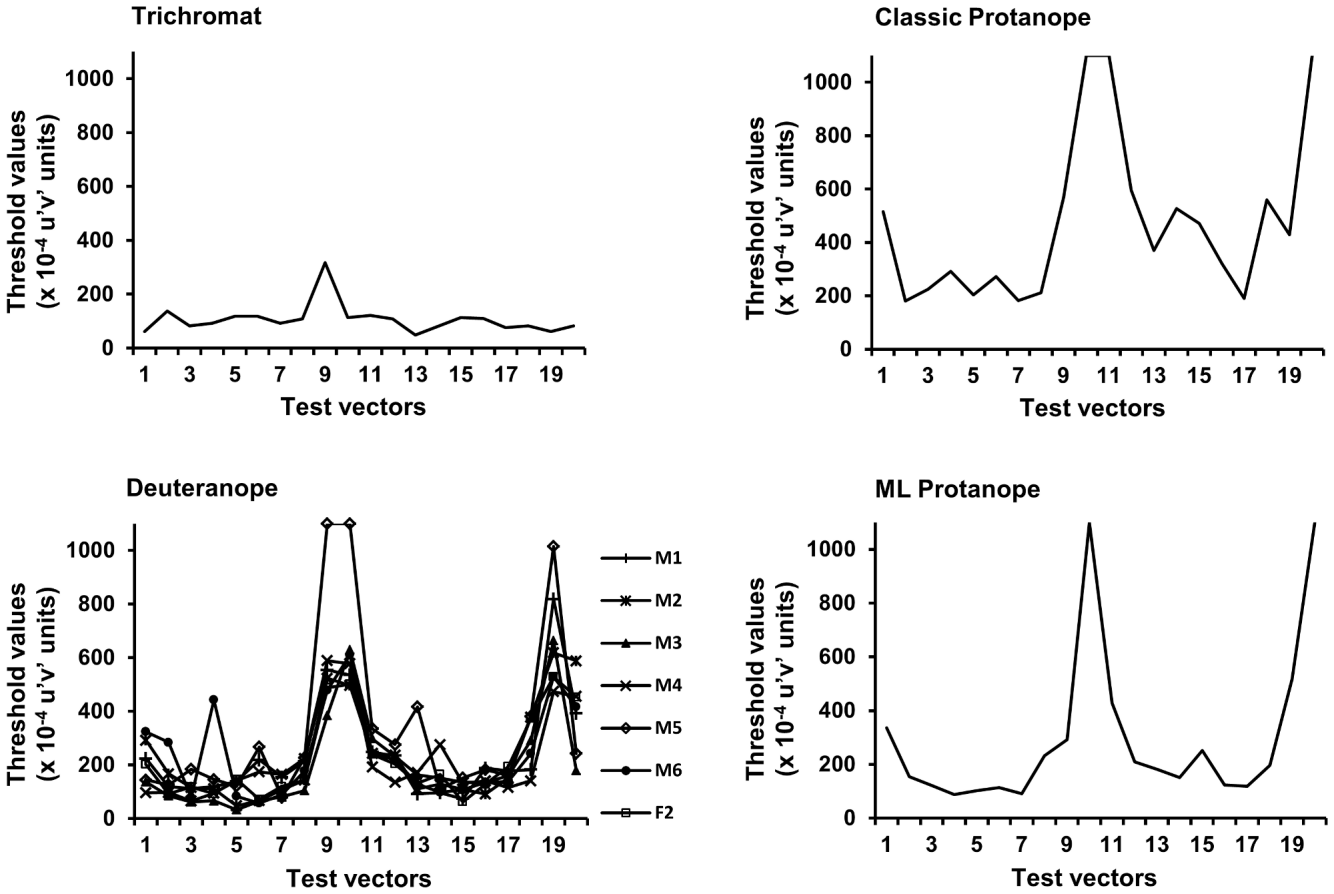
Color discrimination thresholds obtained for capuchin monkeys. The figure shows the thresholds obtained for 20 vectors around the achromatic point of the CIE 1976 u′v′ chromatic diagram (Background 1). The data were grouped according to the phenotypic classification derived from the genetic analysis.

Trichromatic individuals are expected to produce similar thresholds in all 20 test vectors, whereas dichromats are expected to show considerably higher thresholds in at least two complementary test vectors, corresponding to dominant wavelengths that are indistinguishable from the background [40]. Figure 5 presents the thresholds produced by each subject in the 20 vectors tested for the achromatic background (Background 1). The data were grouped according to the phenotypic classification derived from the genetic analysis. As expected, the thresholds produced by F1 were characteristic of a trichromatic individual. Accordingly, all dichromatic subjects presented high thresholds that exceeded 500×10^-4^ u′v′ units in at least one pair of complementary test vectors (i.e., 9–10 and 19–20 for deuteranopes and 10–11 and 20 for protanopes).

Subtle but important differences were found in the discrimination patterns of the different groups. Deuteranopes found it difficult to distinguish from achromatic background hues with dominant wavelengths between 495 and 505 nm (vectors 9–10) and hues in the complementary nonspectral region. Protanopes failed to discriminate hues with dominant wavelengths between 490 and 495 nm (vectors 10–11) and hues in the complementary nonspectral region. Refer to Figure 2 for the dominant wavelength of each vector tested from the achromatic background.

Notably, most animals in the deuteranope group, with the exception of M6, produced thresholds that were lower than the maximum value in vectors 9–10 and 19. The test vectors may have not completely overlapped with the actual confusion line for this phenotype, causing the region of low discriminability to be detected only as a “residual effect” on adjacent vectors. Additionally, as discussed above, false positives and false negatives eventually occurred, contributing to some of the variation observed in this group. Despite these variations, the pattern of distribution of the thresholds was consistent among all of the SYT individuals.

**Figure 6:**
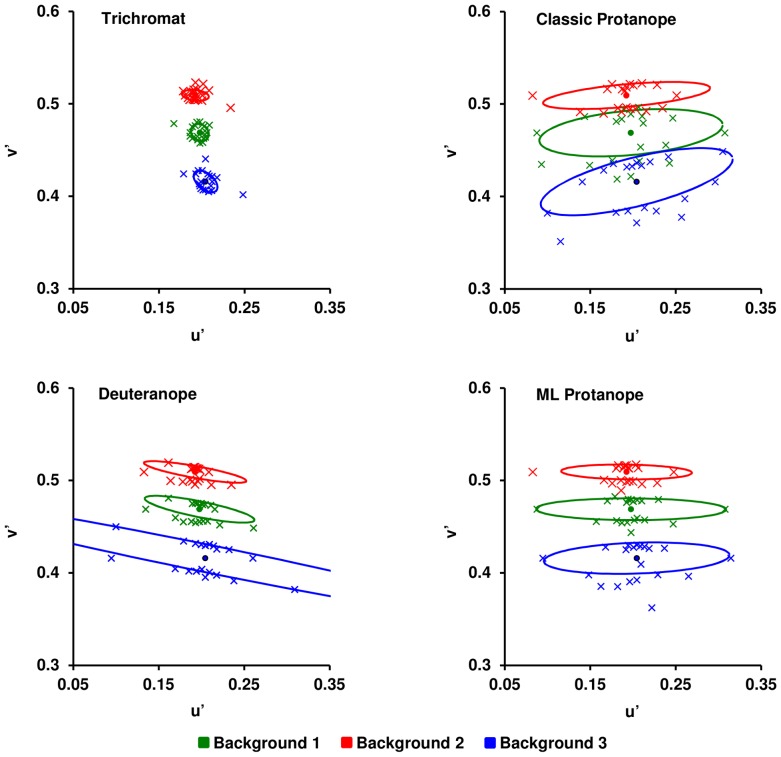
Discrimination ellipses obtained for capuchin monkeys. The figure shows the best-fitting ellipses derived from the thresholds obtained in the 20 test vectors for each background. Deuteranopes are represented by data from subject M4.

Figure 6 presents the discrimination ellipses derived from the complete set of thresholds obtained for the three backgrounds in the CIE 1976 u′v′ diagram. Fitting the ellipses to the data allows quick and intuitive identification of color vision phenotypes because the orientation of the major axes of the ellipses coincides with the orientation of the color confusion lines that is characteristic of each dichromatic phenotype. Subject M4 was randomly singled out as representative of the deuteranope group. For deuteranopes and the “classic” protanope subjects, the orientation of the ellipses was consistent with the phenotypes predicted from the genetic analysis. Notably, although M4 did not produce thresholds with the maximum value in two of the tests, the global distribution of the subject's thresholds was clearly consistent with a deuteranope phenotype.

Interestingly, the orientation of the ellipses differed for the two genetically defined protanopes, with the three lines characteristically converging in the “classic” phenotype but horizontally oriented and parallel to each other in the “ML” phenotype. Such differences suggest behavioral separation between the “classic” and “ML” phenotypes that was not immediately apparent from the analysis shown in Figure 5. This behavioral differentiation of the two protanope phenotypes is consistent with the ERG data that guided the classification proposed by Soares et al. [42], which was adopted in the present study. These authors adopted a Relative Sensitivity Index (RSI) for red and green stimuli, which allows a distinction between deuteranopes and protanopes. Relative Sensitivity Index values less than −0.2 correspond to deuteranopes (SYT), whereas RSI values greater than 0.4 characterize protanopes (AFA). Relative Sensitivity Index values between −0.3 and 0.4 correspond to trichromacy and intermediate sensitivity between “green” and “red” (ML protanope). The two dichromats with the ML cone (AFT) identified in Soares et al. [42] showed RSI values of 0.12 and 0.15, whereas the two protanopes (AFA) showed RSI values of 0.53 and 0.58.

## Conclusions

The present study demonstrated the efficacy of an adapted version of the Mollon-Reffin color discrimination test for application with capuchin monkeys. The Mollon-Reffin test paradigm was originally developed [43]–[45] and validated [46] for the rapid determination of color discrimination thresholds in adult human observers and is used to assess chromatic losses in several different diseases [47]–[58]. Its potential for use with animals had already been demonstrated by Mancuso et al. [40] with squirrel monkeys (*Saimiri* sp.). The adapted stimulus display used in the present study was previously validated against the commercially available version of the Mollon-Reffin test (i.e., the Cambridge Colour Test) in a study that demonstrated high agreement between independent measures made with the original and adapted versions with human adults [39]. Some aspects of the training and testing protocol used in the present study have been shown to be effective for the determination of color discrimination thresholds in 2- to 7-year olds [39].

When applied to capuchin monkeys, the adapted version of the Mollon-Reffin discrimination test demonstrated the expected behavioral result of dichromacy in males and possibility of trichromacy in females and was sufficiently sensitive to differentiate between four phenotypes, in complete agreement with the genetic data. The clear separation between protan and deutan human subjects has already been proven to be a feature of the Mollon-Reffin test paradigm [45]. The replication of this capability with another species, together with the demonstration of a discrimination pattern that is intermediate between protan and deutan by a dichromat that possesses a photopigment with intermediate spectral sensitivity (i.e., between the spectral sensitivity of M and L cones), provides additional evidence of the test's diagnostic refinement.

Given the traditional dichotomic classification of red-green dichromats between deuteranopes and protanopes and their characteristic sets of confusion lines, the behavioral differentiation between “classic” and “ML” protanopes is noteworthy because it suggests identifiable behavioral consequences of even slight variations in spectral sensitivity of M/L cones in dichromats. Perhaps this finding will encourage further investigations of the behavioral implications of the spectrally variant subtypes of cones that are usually averaged as M or L cones in the literature on human color vision [59]–[61].

Reliable information regarding the discrimination capacities of capuchin monkeys may reveal practical implications of genetic or physiological differences observed among individuals. For example, Soares et al. [42] described a possible fourth photopigment for the genus *Sapajus* in female tufted capuchin monkeys (identified as *Cebus apella*), with a spectral sensitivity peak near 552 nm, based on the observation of a new amino acid combination at positions 180, 277, and 285 of the opsin: serine-phenylalanine-threonine (SFT). The existence of a fourth photopigment increases the number of possible color vision phenotypes from six to 10 (four dichromatic, six trichromatic). One important question raised by this finding is whether each different photopigment combination produces correspondingly different behavioral function.

Unfortunately, the sample of animals that participated in the present study did not include any individual with the SFT genotype. Because the absorption peak inferred for the corresponding photopigment (546–553 nm) overlaps with the absorption peak inferred for the AFT genotype (542–547 nm), predicting that the animals with these genotypes will not differ behaviorally is plausible. Given its demonstrated sensitivity to subtle variations in performance, the Mollon-Reffin test may be an ideal tool for investigating whether this is indeed the case.

## Acknowledgments

We would like to thank Ciléa Mesquita, Fabiane Pereira, Juliane da Costa, Rubilene Borges, and Tamyres Roberta Leal for their assistance with data collection. We thank Klena Sarges for veterinarian assistance and Adilson Pastana for the excellent homecage care given to our monkeys. We also thank Givago Souza, Leonardo Dutra, and Marcio Bandeira for their assistance with the selection of ellipse-fitting procedures.
